# Developing a shared understanding of translational science within CTSA hubs through facilitated retreats: A case study

**DOI:** 10.1017/cts.2024.487

**Published:** 2024-02-26

**Authors:** Kristine M. Glauber, Amalia A. Turner, Jessica Sperling, F. Joseph McClernon

**Affiliations:** 1 Clinical and Translational Science Institute, Duke University School of Medicine, Durham, NC, USA; 2 Department of Psychiatry and Behavioral Sciences, Duke University School of Medicine, Durham, NC, USA

**Keywords:** Organizational learning, science facilitation, translational science, team science, workforce development

## Abstract

Translation of critical and broadly impactful health advancements is stymied by insufficient scientific scrutiny of barriers and roadblocks in the process. The Clinical & Translational Science Award (CTSA) funding opportunity announcement released in July 2021 makes clear the distinction between translational research and translational science (TS) and urges a shift from the former to the latter. This represents a significant shift in the overall scientific direction of the CTSA program and necessitates corresponding shifts in CTSA hub operations. To better support TS, the Team Science Core of the Duke CTSA hub designed and facilitated a virtual retreat for hub personnel that (1) enabled organizational learning about TS and (2) identified anticipated challenges and opportunities. A post-retreat survey was utilized to assess the degree to which the retreat met its stated goals. Our survey received a 62% response rate; 100% of respondents would recommend the session to others. Respondents also reported gains in all areas assessed, with evidence for greater understanding of TS and increased perspective of the value and relevance of TS. In this paper, we provide a roadmap for designing and implementing facilitated TS retreats, which we argue is a key step in TS capacity building through workforce development.

## Introduction

Clinical and Translational Science Awards (CTSAs) have historically supported translational research (TR) projects focused on applying basic science discoveries to the development of novel treatments and clinical practices. However, while basic biomedical science has progressed significantly under the CTSA program, these advances have too infrequently translated to greater success in bringing “more treatments to all people more quickly” [1]. As an example, at the current average approval rate of around 50 novel drugs per year [2], it will take at least 190 years to have treatments for the more than 10,000 known human diseases [3].

In its 2021 Funding Opportunity Announcement (FOA), the National Center for Advancing Translational Sciences (NCATS) promoted a fundamental shift in direction for the CTSA program, moving the focus of the award away from TR and to translational *science* (TS) [4]. TS, as a field, aims to accelerate the pace of translation by generating “scientific and operational innovations that overcome the long-standing barriers along the TR pipeline” [5] The FOA makes clear the distinction and rationale for the shift, emphasizing that while TR focuses on “the specific case of a target or disease,” TS is “focused on the general case that applies to any target or disease;” as such, “advances in translational science will increase the efficiency and effectiveness of TR [6].”

Operational changes associated with a shift in the mission of any organization may be impeded by communication disconnects and team misalignment. In the case of CTSA hubs, development and effective implementation of TS-supportive programing can be hampered by a lack of shared understanding of the definitional differences between TR and TS. The two terms have been used interchangeably for decades (e.g., the Clinical & Translational Science Awards themselves supported TR, not necessarily TS). This speaks to the need to educate multiple constituencies (e.g., hub faculty and staff, broader institutional research community, and nonacademic community partners) on the definition and benefits of TS. Our CTSA hub determined that a critical first step in shifting our operational focus would be to develop a common language and understanding of this new field.

Within the CTSA hub structure, Team Science Cores enable effective team formation and functioning (i.e., communicating and integrating diverse perspectives) in a knowledge-producing setting [7]. Further, the Team Science Core at the Duke CTSA hub offers science facilitation services that bring together boundary-spanning teams to address complex scientific questions or societal problems. Science facilitation, an emerging area of practice, accounts for the intellectual, interpersonal, and logistical elements of collaboration to guide teams or groups in the process of integration and collective decision-making [8]. Here we describe methods and outcomes of a retreat that applied science facilitation and Science of Team Science approaches. It serves as an example and framework for facilitating TS activities by educating the workforce and by establishing a shared terminology and working knowledge of TS among a subgroup of hub personnel.

## Methods

### Retreat planning

**Figure 1.**
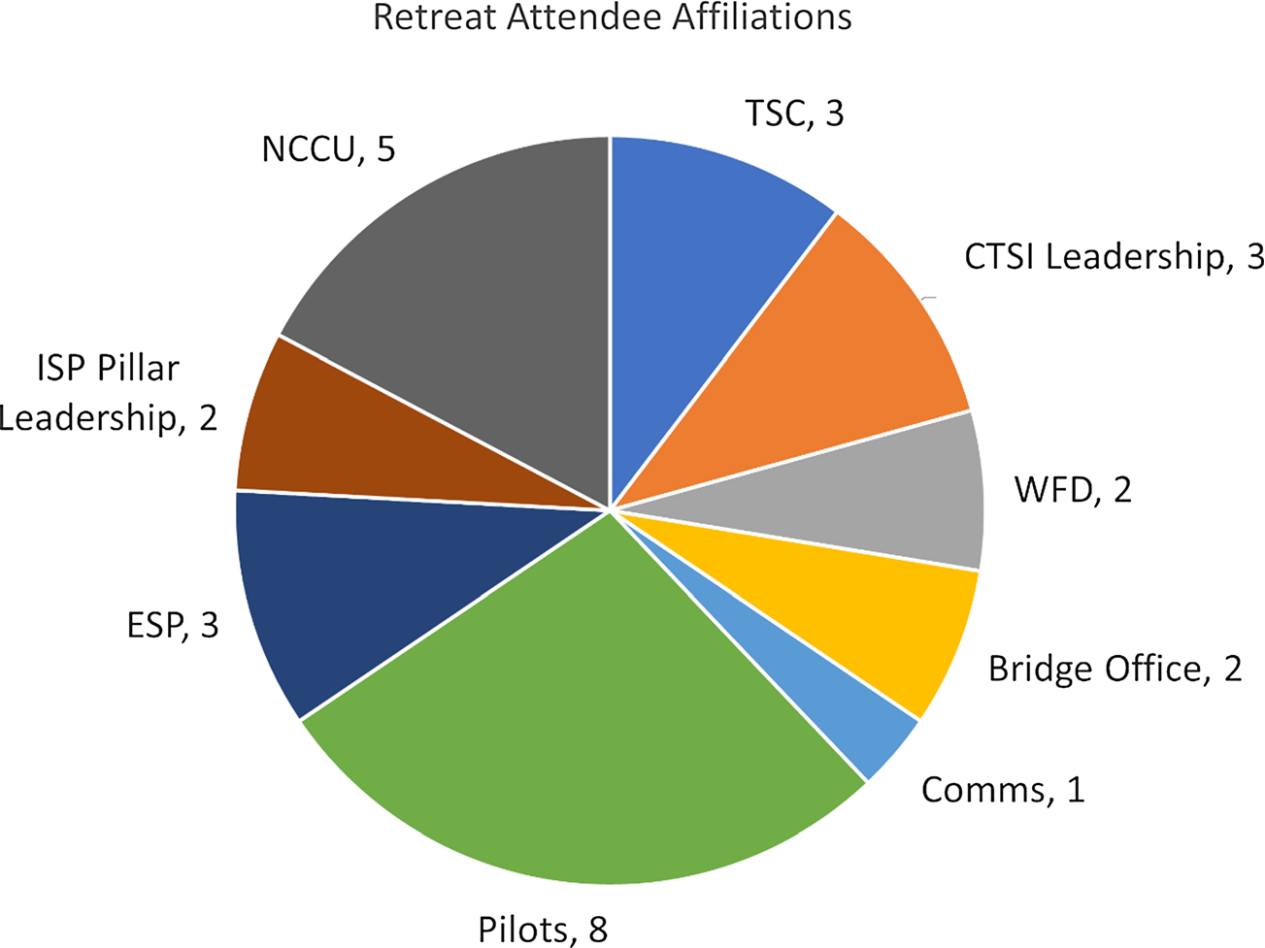
Retreat attendee affiliations. Attendee counts and their programmatic affiliations are shown. Attendees were members of an internal “Integration and Strategic Partnership” (ISP) subgroup of the Duke CTSA, including ISP Leadership (ISP Pillar Leadership), Team Science (TSC), Pilots (Pilots), Evaluation and Strategic Planning (ESP), and the Duke-NCCU Bridge Office (Bridge Office), which functions as the operational link between the Duke CTSI and our partner, North Carolina Central University. Additional invitees included members of our hub Leadership (CTSI Leadership), Communications Core (Comms), Workforce Development Core (WFD), and members from our partner institution (NCCU).

The Team Science Core of the Duke University CTSA hub, in collaboration with hub leadership, designed and executed a facilitated retreat with the aim of assessing, increasing, and aligning organizational understanding of TS [9]. Staff and faculty representatives from several hub cores and programs and from one of our institutional partners (North Carolina Central University; NCCU) were invited to attend the retreat (Fig. 1). Invitees were selected first and foremost as members of an internal “Integration and Strategic Partnership” subgroup of the Duke CTSA. These invitees were selected due to their crucial roles in supporting the shift to translational science focus. Hub leadership, the Team Science Core, and members of the Pilots Core collectively identified the following goals for the retreat: (1) lay the foundation for developing a shared understanding and working knowledge of TS, and (2) surface and identify challenges and uncertainty related to TS understanding and the role of our CTSA hub in increasing institutional capacity for TS. The Evaluation & Strategic Planning Core was engaged to develop, implement, and analyze a summative assessment. The work described herein (Pro00113340) was determined exempt by the Duke University Health System Institutional Review Board.

### Pre-work assignments

All participants were assigned pre-work (see Table 1) with the goal of assessing and ensuring fundamental understanding of TS among retreat participants prior to the session. Case study materials were developed by NCATS Education Branch with the intent of providing examples of TS in preparation for in-session activities [10]. Pre-work was assigned via email 10 days before the retreat and participants were given one week to return responses to pre-work questions.

**Table 1.** Outline of retreat pre-work assignment

Pre-Work Assignment	Purpose
1. Read the article, “Advancing translational science education,” (Faupel-Badger et al., 2022).	Ensure baseline level of understanding among retreat participants.
2. Answer the questions below and return your responses to facilitators three business days before the retreat: a. Question 1: After reading this article, how do you define translational science (TS)?	Assess participants’ level of understanding.
	Prepare for in-retreat activities.
b. Question 2: What were your main takeaways from the article?	
c. Question 3: List any new-to-you jargon or terms that were used in the article, or terms that had a new and different meaning/context for you and define them.	
d. Question 4: What questions do you still have after reading this article?	
3. Watch the TS Case Study Video (one of the following videos from the NCATS Translational Science Principles series was randomly pre-assigned to each attendee):	Prepare for breakout room discussions.
a. Partnering with Patient Advocates to Advance Research on All Rare Diseases, or	
b. Metarrestin for Cancer Metastasis, or	
c. Developing Tissue Chips to Transform Drug Discovery and Development.	

Pre-work responses were anonymized, compiled, and analyzed by the facilitation team for use during the session. Multiple approaches were used to integrate pre-work responses into retreat activities. The website, TagCrowd.com, was used to determine word-use frequencies in Question 1 responses. Facilitators also employed open coding to systematically review and categorize the textual data from pre-work responses. Codes were not predetermined but emerged from the data itself. The initial open codes captured the key concepts in the responses to Questions 2 and 4 to better determine how the article influenced participants’ understanding of TS and identify components of the article for which participants sought further understanding. Finally, Google Jamboard, a collaborative whiteboard technology, was used to represent terms identified by respondents in Question 3 (see “Pre-work summary and discussion”).

### Retreat protocol

The retreat was conducted virtually in two parts separated by a 15-minute break (See Supplement, Figure S4 for a detailed agenda). Part 1 (60 minutes) included 25 minutes for a welcome, introductions, an overview of retreat goals and agenda, and an icebreaker activity, followed by 35 minutes dedicated to review and discussion of pre-work themes. Part 2 (75 minutes) included 65 minutes for breakout and whole group discussion of TS case studies and 10 minutes for final remarks and next steps.

#### Introductions and framing of retreat goals

Team Science Faculty Co-Director (FJM) welcomed attendees and shared the purpose of retreat activities. Team Science Core staff (KG, AT), who fulfilled the role of facilitators introduced themselves and communicated retreat ground rules and norms of interaction. Emphasis was placed on encouraging active engagement by (1) asking participants to turn on their video and (2) introducing the concept of psychological safety and stressing its importance in a team-learning setting. Finally, facilitators led an icebreaker activity, which was adapted from Heberger-Marino and Stephens to build trust and camaraderie through identification of shared interests and experiences [11].

#### Pre-work summary and discussion

Synthesized pre-work responses were shared with participants via PowerPoint and Google Jamboard, the latter of which was used throughout the retreat in multiple ways (Table 2). We used Jamboard to (1) capture outstanding questions, (2) display and sort topics of discussion from the pre-work responses, and (3) record ideas and call attention to outstanding needs. Jamboard content created during the retreat was collected and sorted by facilitators and summarized in the Results. Participants were then invited to ask questions and discuss the results. Facilitators provided a gentle redirect when discussion became focused on a single domain of hub operations (e.g., criteria for evaluating TS pilot projects).

**Table 2.** Google Jamboards created for the retreat

Jamboard Title	Description
Retreat Parking Lot	An in-retreat-generated repository for ideas, questions, and concerns that participants felt warranted consideration but were outside of the scope of the current retreat.
Shared Lexicon of Translational Science (TS)	A repository of terms identified as new to participants or needing further clarification. Included jargon and other terms identified from pre-work responses and those identified during the retreat.
Pre-work Remaining Questions	A pre-populated board containing submitted responses to Question 4 of the pre-work (see Table 1, Section 2 for questions).
TS Case Study Discussion	An in-retreat-generated board to visualize and record large-group discussion of the three case study videos.
TS Greatest Opportunities	An in-retreat-generated board to record participants’ perceptions of the greatest opportunities offered by a shift in focus to TS.
TS Greatest Challenges	An in-retreat-generated board to record participants’ perceptions of the greatest challenges posed by a shift in focus to TS.

Shown are the titles and descriptions of each collaboratively produced Jamboard.

#### Case study discussions

Participants were divided into breakout rooms and given prompts to guide discussion (20 minutes) of their assigned TS case study through the lens of the eight key principles for effective TS [6]. The use of case studies is important in the context of this retreat as the audience included faculty and staff who are involved in developing and implementing TS-supportive programing. Following the breakout room discussion, participants returned and a rapporteur from each group provided an overview of their assigned case study, identified the TS elements of the project, noted any issues that came up during discussion, and recorded ideas and questions inspired by the case studies. A large-group discussion based on breakout group report-outs followed. The final retreat activity employed pseudo-Nominal Group Technique [12] and Jamboards to identify and record the single greatest opportunity and challenge posed by a shift in focus to TS.

### Post-retreat evaluation

A post-retreat Qualtrics survey assessed TS knowledge change (e.g., ability to recognize a TS project; confidence in answering questions about TS), and change in attitude or perspective (e.g., perceived value of TS; perceived relevance of TS to ones’ current or future work). Participants rated each statement on a five-point Likert-type scale reflecting how true the statements were for them before and after the session. Open-ended questions prompted participants to describe the effect of the retreat and ongoing concerns about TS.

Experience-focused questions addressed engagement in the retreat, comfort in sharing concerns or uncertainties around TS, perspective on retreat length, the value of distinct components of the retreat, and open-ended questions addressing added perspective on experience.

A link to the survey was emailed immediately after, and two additional times following the session. Results were analyzed using descriptive statistics, including frequency and means of response options.

## Results

### Retreat participation

Thirty individuals were invited to participate in the retreat: 24 from the Duke CTSA and 6 from NCCU. Of those, 86% (*n* = 26; Duke = 22; NCCU = 4) attended. Participants included both faculty (38%) and staff (62%). 53% (*n* = 16) of invitees submitted responses to the pre-work questions.

### Pre-work results

Submitted responses to the pre-work are summarized below.

*Question 1: After reading this article, how do you define TS?* Participants held a shared understanding that TS is a framework of operational and scientific principles applied to the process of TR with the goal of improving health outcomes. Illustrative responses included, *“research about how we do the research and how we can do it better”* and *“The systematic study of the process of turning observations in the laboratory, clinic, and community into interventions that improve the health of individuals and the public….* The connection of team science to TS was also recognized, *…[TS] is really team science applied to the TR process.*

*Question 2: What were your main takeaways from the article “Advancing translational science education?”* The following themes were identified:

1. **Defining TS.** The operational and scientific principles of TS are informed by a growing evidence base.
2. **Value of TS.** TS offers the potential to increase speed, efficiency, and equity while reducing costs.
3. **TS education and training.** Education and training in TS are essential to the TS process and to workforce development. Additionally, the curriculum around TS must be continually evaluated and updated to remain current with the evolving state of the science.
4. **TS competencies.** A translational scientist must be more than simply interdisciplinary; they must also be knowledgeable in the translational process.
5. **Further needs.** A successful TS program will require both institutional and cultural change, intentional engagement with the public, a more diversified workforce, and robust evaluation of TS education and training impacts.

In addition to the takeaways from the article, the reading inspired several participants to report action items that would be beneficial to the hub, including conducting surveys to identify those interested or engaged in TS, conducting analyses of journals and funding opportunities that support TS scholarship, conducting multi-case studies, and creating visual aids to help clarify the relationship between TR and TS.

Question 3: “*Please list any new-to-you jargon or terms that were used in the article, or terms that had a new and different meaning/context for you and define them.”* The terms that participants identified are described in the summary of “Jamboard 2: Shared Lexicon of TS” in the next section.

*Question 4: What questions do you still have after reading this article?* Most responses to this question focused on concerns about how TS would be integrated into hub operations. Identified themes and thematic questions included:

1. **TS education and training.** How and at what stage will training in TS be operationalized (i.e., just-in-time with teams, foundational as part of CTSA participation, or part of CTSA pilot proposal creation process)? How do we provide a spectrum of training, depending on the needs of the translational team, and how do we determine whether deep knowledge or a more general familiarity with TS is needed?
2. **Implementation.** How will researchers and institutions be incentivized to engage in and/or promote TS? How can we best help researchers understand and apply the TS framework?
3. **Funding and dissemination.** What opportunities are available to support TS scholarship?
4. **Evaluation.** What metrics should be used to determine hub impact on TS efforts?
5. **Workforce.** How do we identify researchers interested in TS? Will the TS workforce come primarily from TR or from the broader community? How can we offset the opportunity cost of engaging in TS for junior researchers needing to conduct TR? How do we integrate those outside of academic pathways into TS teams? What is the best allocation of resources for supporting TS within the hub?
6. **Community engagement**. What are the ways in which community members and community partners can be involved in the TS process?

Discussion of many of these questions was not within the scope of this retreat. However, facilitators informed participants that their input would be preserved for future consideration and discussion.

### In-retreat results: Jamboard summaries

#### Parking Lot

Participants primarily used the *Parking Lot* to raise questions and voice concerns about TS within the context of the hub and more broadly within the scholarly community. Participants also shared considerations for promoting and supporting TS, including expanding the TS workforce to those not on the academic pathway, framing TS knowledge as a competitive advantage for junior investigators interested in careers in industry, and leveraging evaluation to better determine what constitutes TS. Finally, participants used the *Parking Lot* to highlight terms with which they were unfamiliar or needed more clarity within the context of TS.

#### Shared lexicon of TS

Participants identified terms on this board with established definitions (e.g., multi-case study) and terms that were new to them but clearly defined in the article (e.g., core competencies for clinical and translational research). Discussion focused on the remaining terms (e.g., experiential TS education), which were more nuanced and not well defined in the article.

#### Pre-work remaining questions

Participants added any questions that remained after completing the pre-work assignment to the *Pre-work remaining questions* board. The questions submitted with the pre-work responses are summarized above in the section for pre-work results, Question 4. The only additional question added to the Jamboard was about how the fields of implementation science, team science, and TS overlap.

#### TS case study

Component elements of the three case studies were shared and used to identify common characteristics of TS projects. In the discussion, participants were not always clear about what research counts as TS and how to use the TS principles to determine if a given project qualifies as TS. Participants wondered whether the degree of alignment with the TS principles could be used to determine whether a project is TS. Finally, the group collectively decided that the principle of “generalizable solutions” is the key determinant.

#### TS opportunities

Many participants noted opportunities for innovation afforded by a change in the mission of CTSA hubs to focus on TS, along with the opportunity to identify and address gaps or challenges in TR that currently limit the impact of research on patient health. The opportunity to engage in boundary-spanning collaborations across disciplines, programs, and institutions was also highlighted by many, and some of the participants identified boundary-spanning engagement with the community as an opportunity to address and advance health equity. Finally, a number of participants identified opportunities to help shape the nascent field of TS by establishing best practices and training a diverse workforce in TS.

#### TS challenges

The most common challenges identified revolved around incentivizing the research community to engage in TS and motivating the institution to support TS. Many participants also anticipated challenges in communication, with specific concerns about how to establish and communicate a shared understanding and vision of TS within our hub and beyond. Other challenges included encouraging researchers to engage in equitable, boundary-spanning collaborations; how to provide TS training and education at multiple learner levels; and how to address opportunity costs presented by potentially shifting focus away from TR.

### Post-retreat evaluation

Of the 26 retreat participants, 16 (62%) completed the post-retreat survey.

#### Gains

**Figure 2.**
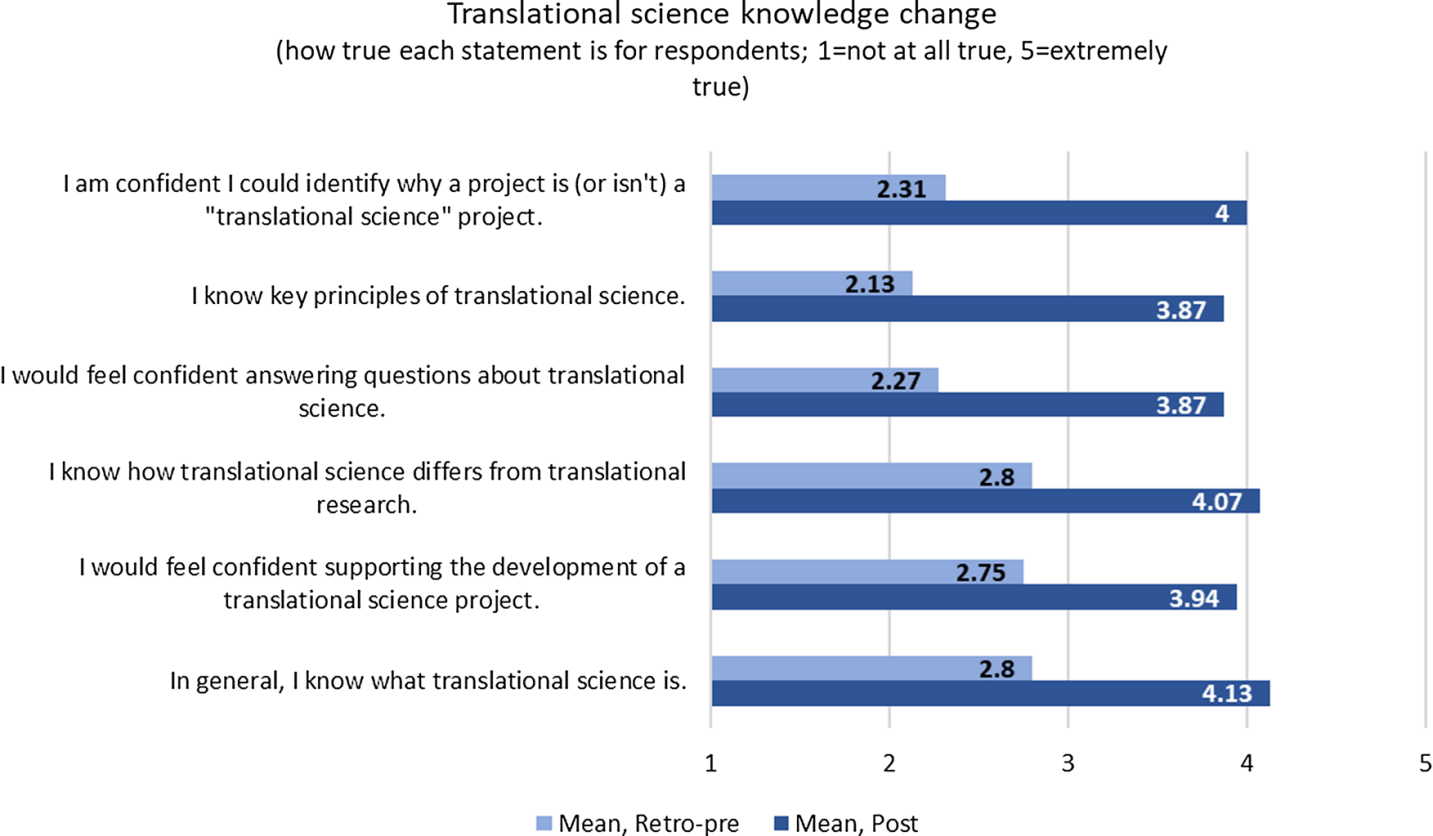
Self-reported translational science (TS) knowledge change. Average participant responses to varied statements related to TS perspective and knowledge before (retro-pre) and after (post) the retreat.

Respondents reported gains in all areas assessed, with evidence for greater understanding of TS (Fig. 2) and increased perspective of TS as valuable and relevant to one’s own work (Supplement, Figure S1).

#### Experience

A majority of respondents found all components very or extremely valuable; the pre-reading article and the introductory slides, however, were relatively less valued (see Supplement, Figure S2). Open text responses (Supplement, Table S1) indicated the central value of discussion (“*Breaking out in the groups and discussing case studies really helped me to better understand opportunities within TS within “real life” scenarios.”*) and peer engagement (“*Questions and observations from fellow retreat attendees gave me a more nuanced understanding of TS and its potential impacts.”)*.

Overall, 94% of participants (15 of 16) reported they very, or extremely agree they would recommend attending a retreat like the one they attended; one participant somewhat agreed with this statement (Supplement, Figure S3). However, despite a focus on creating a safe forum for sharing, some (2, 13%) indicated hesitancy in sharing their uncertainties about TS. Respondents generally (81%) found the length appropriate, though some (19%) felt it was too long. This was also reflected in open-ended data on desired change, noting that length may have felt more appropriate for an in-person retreat.

#### Ongoing concerns

Open text responses indicated primary concerns centered largely on issues of buy-in, including among investigators at each institution (*“I am still uneasy about engaging researchers and getting them excited about the possibilities”)* as well as institutional leadership (*“[I’m concerned]…that researchers and institutional leadership at our institutions will embrace the idea of doing TS”).*

## Discussion

We developed and implemented a facilitated retreat to build TS workforce capacity, enable shared understanding, and identify barriers to implementing TS-supportive programing within our institution. As CTSA hubs around the nation pivot the focus of their scientific support efforts to TS, our work indicates that facilitated retreats can be a resource-efficient and effective method for engaging hub personnel on the topic of TS.

Building on organizational learning theory [13], we hypothesized that a facilitated retreat would enable effective learning within a subgroup of our hub [14]. We found that knowledge of TS, as well as perceived confidence in supporting TS projects, increased because of our workshop. Our method for conducting a facilitated co-learning retreat was an effective means to socialize and clarify the concept of TS. Participants reported that the format and components of the interactive retreat contributed positively to their understanding of TS, and our retreat helped participants feel confident bridging the “know-do” gap to complete their future job duties, e.g., in identifying TS projects, answering questions about TS, and supporting the development of TS projects. These findings are consistent with the definitional purpose of facilitation- namely that “*[f]acilitation is a goal-oriented, context-dependent social process for implementing new knowledge into practice or organizational routines*” [14].

Further, our facilitated approach enabled higher-order organizational learning [15], specifically providing not only insight into perceived challenges in supporting TS but also the co-development of solutions to overcome these challenges. Enabling this kind of higher-order learning is important for organizational resilience in uncertain environments [16], such as during a hub’s transition to supporting TS. The organizational change faced by CTSA hubs in response to the July 2021 FOA will take time, strategic assessment of critical environmental factors of success, and utilization of evidence-based methods and processes to achieve the desired outcome. Structured facilitation and its theoretical and methodological disciplinary home of Integration and Implementation Sciences may be useful in effecting this change [16].

### Limitations and future directions

Our work suggests that facilitated retreats may be an effective means of individual and organizational learning about TS; however, the authors acknowledge the limitations of drawing conclusions from case studies such as this one. First, response rates to pre-work questions and post-retreat evaluations were less than optimal. In order to increase response rates we plan to streamline retreat pre-work (e.g. condense required readings and reduce pre-work questions) and add a 5-minute post-retreat survey period to the end of the retreat. Second, our retreat was planned and run by internal facilitators. In future work, we will evaluate whether comparable outcomes are achieved with external facilitators. Third, a major factor in what made our retreat successful was the support and unifying messaging from leadership, both in their assessment of the necessity of a collaborative solution to address this challenge and in their confidence in the Team Science Core to design and execute the activity. Current work by our team is investigating the disseminability of the retreat to other hubs.

Our work revealed areas for continued focus at our hub and likely beyond in implementing TS-supportive programing. First, despite an effort on the part of NCATS to clarify differences in the meaning and scope of TS, significant variability in understanding remains. Second, our post-retreat evaluation survey identified that personnel feel uncertain about the operationalization of TS support within our hub, and more broadly, how to socialize and incentivize engagement in TS among our research community and partners. We plan to run more facilitated co-learning retreats within our hub, institution, and in collaboration with partners (e.g., community and other hubs) as a means of addressing these challenges. The authors invite further discussion from CTSA and other colleagues with an interest in a scholarly community to support TS at an institutional level.

## Supporting information

Glauber et al. supplementary material Glauber et al. supplementary material

## Supplementary material

The supplementary material for this article can be found at https://doi.org/10.1017/cts.2024.487.

## References

[1] Austin CP. Opportunities and challenges in translational science. Clin Transl Sci. 2021;14(5):1629–1647. doi: 10.1111/cts.13055. 33982407 PMC8504824

[2] Mullard A. 2022 FDA approvals. Nat Rev Drug Discov. 2023;22(2):83–88. doi: 10.1038/d41573-023-00001-3. 36596858

[3] National Center for Advancing Translational Sciences. Speeding More Treatments for All 2023;2023. https://ncats.nih.gov/sites/default/files/NCATS_Speeding-Treatments-Fact-Sheet_508.pdf. Accessed November 15, 2023.

[4] National Center for Advancing Translational Sciences. Clinical and Translational Science Award (UM1 Clinical Trial Optional), https://grants.nih.gov/grants/guide/pa-files/PAR-21-293.html. Accessed January 30, 2024.

[5] Rutter JL. How NCATS Tackles Persistent Problems in Translation, 2023. https://ncats.nih.gov/director/june-2023. Accessed November 15, 2023.

[6] Faupel-Badger JM , Vogel AL , Austin CP , Rutter JL. Advancing translational science education. Clin Transl Sci. 2022;15(11):2555–2566. doi: 10.1111/cts.13390. 36045637 PMC9652430

[7] Pelfrey CM , Goldman AS , DiazGranados DJ. What does team science look like across the CTSA consortium? A qualitative analysis of the great CTSA team science contest submissions. J Clin Transl Sci. 2021;5(1):e154. doi: 10.1017/cts.2021.812. 34527293 PMC8411266

[8] Cravens AE , Jones MS , Ngai C , Zarestky J , Love HB. Science facilitation: navigating the intersection of intellectual and interpersonal expertise in scientific collaboration. Humanit Soc Sci Commun. 2022;9(1):256. doi: 10.1057/s41599-022-01217-1.

[9] Kaner S. Facilitator’s Guide to Participatory Decision-Making. 3rd ed. San Francisco, CA: John Wiley & Sons; 2014.

[10] National Center for Advancing Translational Sciences. Translational Science Principles, https://ncats.nih.gov/about/about-translational-science/principles. Accessed December 4, 2023.

[11] Heberger-Marino A, and Stephens, Sarah. Creating Conditions for Playful & Mindful Facilitation, 2023. https://www.intereach.org/webinararchives/2021/9/23/september-14thnbspcreating-conditions-for-playful-amp-mindful-facilitationnbsp. Accessed December 4, 2023.

[12] Powers CM , Dana G , Gillespie P , et al. Comprehensive environmental assessment: a meta-assessment approach. Environ Sci Technol. 2012;46(17):9202–9208. doi: 10.1021/es3023072. 22889372 PMC3439956

[13] Argote L , Miron-Spektor E. Organizational learning: from experience to knowledge. Organ Sci. 2011;22(5):1123–1137.

[14] Berta W , Cranley L , Dearing JW , Dogherty EJ , Squires JE , Estabrooks CA. Why (we think) facilitation works: insights from organizational learning theory. Implement Sci. 2015;10(1):141. doi: 10.1186/s13012-015-0323-0. 26443999 PMC4596304

[15] Duchek S. Organizational resilience: a capability-based conceptualization. Business Res. 2020;13(1):215–246. doi: 10.1007/s40685-019-0085-7.

[16] Schuttner L , Coleman K , Ralston J , Parchman M. The role of organizational learning and resilience for change in building quality improvement capacity in primary care. Health Care Manage Rev. 2021;46(2):E1–e7. doi: 10.1097/hmr.0000000000000281. PMC7541444 33630509

