# Supplementary material for: Developing a shared understanding of translational science within CTSA hubs through facilitated retreats: A case study
Source: J Clin Transl Sci. 2024 Feb 26;8(1):e49. doi: 10.1017/cts.2024.487 (PMC10951925; doi:10.1017/cts.2024.487)
Supplement: Glauber et al. supplementary material [file S2059866124004874sup001.pdf]

Supplementary materials

Figure S1. Translational science attitude change

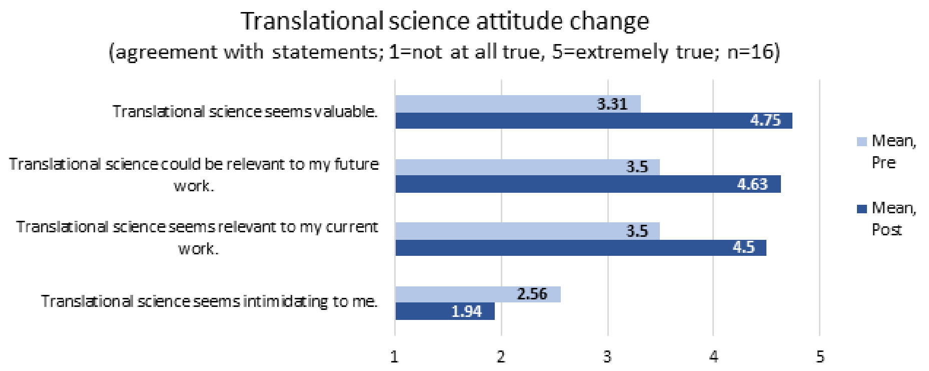

Figure S2. Value of Retreat Components to understanding of Translational Science

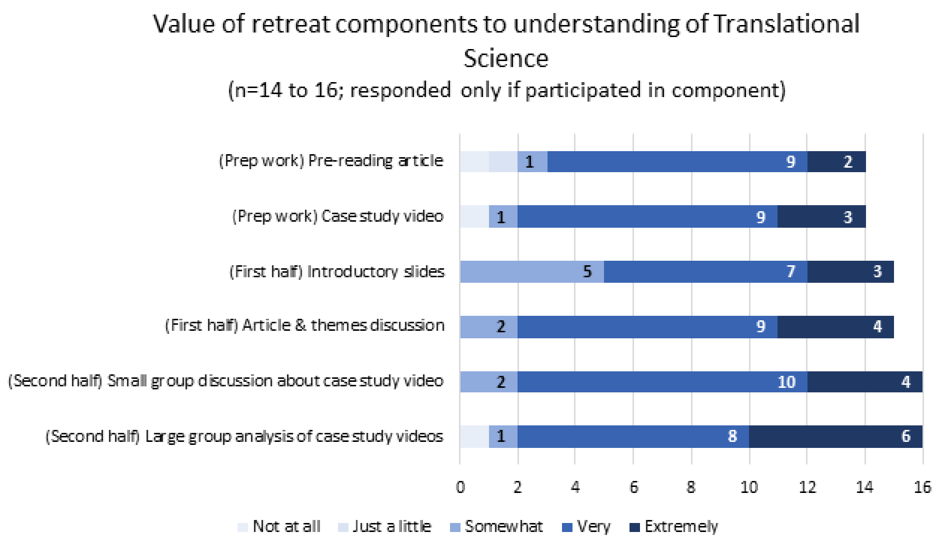

Figure S3. Overall retreat reflection

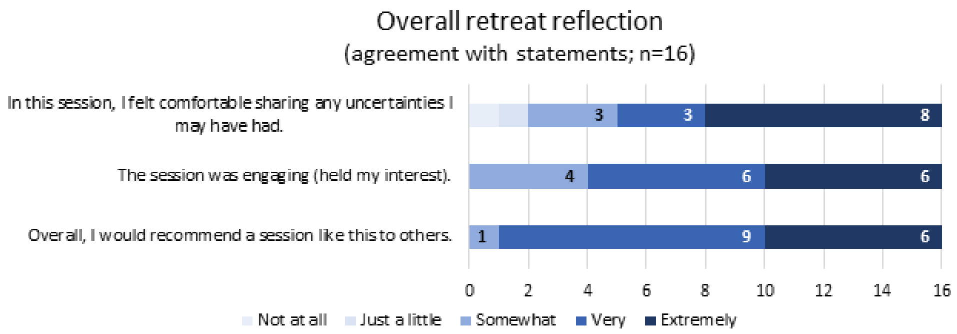

Table S1. Open-text responses to post-retreat survey questions

Topic	Example responses
Effects/relevance of translational science	
	Exploring the multi-faceted impacts of translational science, above and beyond creating efficiencies, was interesting and motivating.
	Relevance [of translational science] to CTSI/CTSA work going forward.
TS connections to other arenas/topics	
	Understanding that the process begins with a research question (translational research) that could turn into a generalizable finding (translational science) is what we need to focus on.
	Parallels between translational science and team science, and how those two elements work so well together

Theme	Example response
Component/elements of retreat (largely case studies and small group discussions)	
	Small group discussion on case studies
	Small group discussions
	Breaking out in the groups and discussing case studies really helped me to better understand opportunities within translational science within "real life" scenarios.
	Walking through the distinctions and interpretations of the terms e.g., the

	example of the definition of Vectors
	The generalizable takeaways and differentiation between translational research and translational science were most valuable for me.
	Skillful facilitation
Peer engagement / exchange	
	Questions and observations from fellow retreat attendees gave me a more nuanced understanding of translational science and its potential impacts.
	Hearing perspectives from others, especially those who are from NCCU
	The clarifying questions and responses related to translational science
	Clarifying discussions on the operationalization of the constructs.
	thoughtful discussion
Different perspectives	
	Many different ways to look at translational science.
Discussion with colleagues (related to peer engagement/exchange, but focused on discussion with others from CTSI)	
	Just having dedicated time to discuss and delve into translational science (the concept itself), and being able to do so with others in the CTSI world.
	Connecting with my colleagues and sharing ideas about translational science, specifically the opportunities and hurdles that we will face in the future.

**Them e**	**Example Quote**
Buy-in / interest	
	[I] know there will be resistance to the changes needed to look at the bigger picture.
	Getting people on board with the process.
	That researchers and institutional leadership at our institutions will embrace the idea of doing translational science
	I think understanding and buy-in will continue to be a bit challenging
	I am still uneasy about engaging researchers and getting them excited about the possibilities. I am also uneasy regarding the amount of pilot funding that we can dedicate to these awards- it is hard to get PIs excited about $25-50k.
Education/understanding needs	
	How to educate trainees
	That we can accurately and compellingly articulate what translational science is, why it matters, and what our fundamental shift is (within the CTSI) in our approach to supporting translational science at Duke
"Grand" nature	
	I am concerned people will impose individual aspects on translational

	science projects and turn away investigation from Translational Science with high hurdles or expectations rather than reviewing against Translational Science steps.

Figure S4. Detailed retreat agenda

Translational Science Retreat

Session Goals:
1) To lay the foundation for developing a shared understanding and working knowledge of translational science (TS), and

2) To surface and identify challenges and uncertainty related to TS understanding and the role of our CTSA hub in increasing institutional capacity for TS.

Information Sharing | Energizer/Icebreaker | Exercise | Discussion/Debriefing | Break

TIME	NAME	DESCRIPTION	ADDITIONAL INFO
		**First session**	
00:00<br>5m	**Welcome**	Pillar leadership welcomes participants and frames the retreat.	Speaker frames the retreat by providing their perspective on the following questions:<br>• What is the problem we are addressing?<br>• Why are we addressing it now?<br>• What is our vision for our work together?<br>Visual aid will be provided in the form of a slide deck.
00:05<br>5m	**Introduction**	Retreat facilitators (KG and AT) introduce themselves, review the retreat goals and establish norms for interaction during the retreat.	Norms for interaction:<br>• Please turn your video on, but feel free to turn your video off and step away to attend to other obligations in your workspace, as needed.<br>• Everyone's voice is equally important and helpful. We want to hear from everyone during the discussion today, but you don't have to answer every question.<br>• There are no right or wrong answers to the questions posed. Remember to say what is true for you.<br>• Only one person speaks at a time so we can hear everyone's comments. Please put yourself on mute while others are speaking.<br>• Please silence your electronic devices and notifications during the meeting.<br>• Please use the Jamboard "Parking Lot" to capture any items that need to be addressed (e.g., outstanding questions or needs, and jargon)
00:10<br>15m	**Icebreaker: "I share common ground with..."**	Facilitators introduce and lead an activity to build connections among participants and foster engagement.	Instructions:<br>1. To start, all participants turn off their video and mute their audio feeds<br>2. For the first round, Facilitator states "I share common ground with people who [insert interest or attribute]."<br>3. Retreat participants who agree with the statement turn on their video feed, allowing participants to build trust and camaraderie around shared interests.<br>4. Facilitator who shared the statement in the first round then chooses another participant to share among those who turned on their video feed.<br>5. This activity was repeated for several rounds allowing for many participants to share their interests.
00:25<br>15m	**Pre-work analysis summary**	Facilitators use prepared slides to review themes identified in submitted pre-work responses.	
00:40<br>20m	**Facilitated Discussion**	Participants respond and ask questions regarding the content shared by pillar leadership and the facilitators.	Facilitators provided a gentle redirect when the discussion occasionally moved into topics not germane to the goals of the retreat - such as translational science operationalization and implementation - and encouraged input from as many participants as time would allow.
01:00<br>15m	**Break**		
		**Second Session**	
01:15<br>5m	**Introduction of Breakout Group Activity**	Participants reorient to the agenda and the activity for the second session: case study breakout groups.	Groups randomly pre-assigned to watch one of the three translational science case study videos prepared by NCATS.

TIME	NAME	DESCRIPTION	ADDITIONAL INFO
			1. Partnering with Patient Advocates to Advance Research on All Rare Diseases, or<br>2. Metarrestin for Cancer Metastasis, or<br>3. Developing Tissue Chips to Transform Drug Discovery and Development.
01:20<br>30m	**Breakout Rooms: Case Study Discussion**	Group participants discuss the case study using the provided discussion points.<br>Participants introdcue themselves before speaking.<br>A rapporteur will be chosen to report key discussion points for the following large group discussion.	Case study discussion points:<br>1. Describe the case study in one sentence.<br>2. Using the 8 Translational Science Principles identified in the reading (Faupel-Badger et al. 2022) indicate whether each principle was met in this case study and describe the aspects of this project that met each principle (please feel free to refer to Table 1 from the reading for definitions of each principle).<br>3. Could this template be a useful tool for your team in supporting the development and evaluation of a translational science proposal?<br>4. What other tools or questions would be useful in evaluation a proposal from a translational science perspective?
01:50<br>25m	**Report-outs and Large Group Discussion**	Participants return to main room. Rapporteurs from each group share insights from their group's discussion.<br>Short facilitated discussion.	Facilitators document common elements of case studies in Jamboard.
02:15<br>10m	**Opportunities and Challenges**	Participants share their vision for future shared work in response prompts via Pseudo-Nominal Group Technique.	Prompts:<br>1. From your vantage point, what is the greatest opportunity this shift in focus to translational science affords us?<br>2. From your vantage point what is the greatest challenge facing us with the shift in focus to translational science.<br>Participants submit responses in Jamboard.
02:25<br>5m	**Concluding remarks and next steps**	The session closes with a few words from Pillar leadership and an overview by the facilitators and leadership about anticipated next steps.	

02:30

TOTAL LENGTH: 2h 30m

Translational Science Retreat - block details

First session

00:00
5m

Welcome

Pillar leadership welcomes participants and frames the retreat.

ADDITIONAL INFORMATION

Speaker frames the retreat by providing their perspective on the following questions:

- What is the problem we are addressing?
- Why are we addressing it now?
- What is our vision for our work together?

Visual aid will be provided in the form of a slide deck.

00:05
5m

Introduction

Retreat facilitators (KG and AT) introduce themselves, review the retreat goals and establish norms for interaction during the retreat.

ADDITIONAL INFORMATION

Norms for interaction:

- Please turn your video on, but feel free to turn your video off and step away to attend to other obligations in your workspace, as needed.
- Everyone's voice is equally important and helpful. We want to hear from everyone during the discussion today, but you don't have to answer every question.
- There are no right or wrong answers to the questions posed. Remember to say what is true for you.
- Only one person speaks at a time so we can hear everyone's comments. Please put yourself on mute while others are speaking.
- Please silence your electronic devices and notifications during the meeting.
- Please use the Jamboard "Parking Lot" to capture any items that need to be addressed (e.g., outstanding questions or needs, and jargon)

00:10
15m

Icebreaker: "I share common ground with..."

Facilitators introduce and lead an activity to build connections among participants and foster engagement.

ADDITIONAL INFORMATION

Instructions:

1. To start, all participants turn off their video and mute their audio feeds
2. For the first round, Facilitator states "I share common ground with people who [insert interest or attribute]."
3. Retreat participants who agree with the statement turn on their video feed, allowing participants to build trust and camaraderie around shared interests.
4. Facilitator who shared the statement in the first round then chooses another participant to share among those who turned on their video feed.
5. This activity was repeated for several rounds allowing for many participants to share their interests.

00:25
15m

Pre-work analysis summary

Facilitators use prepared slides to review themes identified in submitted pre-work responses.

ADDITIONAL INFORMATION

00:40
20m

Facilitated Discussion

Participants respond and ask questions regarding the content shared by pillar leadership and the facilitators.

ADDITIONAL INFORMATION

Facilitators provided a gentle redirect when the discussion occasionally moved into topics not germane to the goals of the retreat - such as translational science operationalization and implementation - and encouraged input from as many participants as time would allow.

01:00
15m

Break

Second Session

01:15
5m

Introduction of Breakout Group Activity

Participants reorient to the agenda and the activity for the second session: case study breakout groups.

ADDITIONAL INFORMATION

Groups randomly pre-assigned to watch one of the three translational science case study videos prepared by NCATS.

1. Partnering with Patient Advocates to Advance Research on All Rare Diseases, or
2. Metarrestin for Cancer Metastasis, or
3. Developing Tissue Chips to Transform Drug Discovery and Development.

01:20
30m

Breakout Rooms: Case Study Discussion

Group participants discuss the case study using the provided discussion points.

Participants introdcue themselves before speaking.

A rapporteur will be chosen to report key discussion points for the following large group discussion.

ADDITIONAL INFORMATION

Case study discussion points:

1. Describe the case study in one sentence.

2. Using the 8 Translational Science Principles identified in the reading (Faupel-Badger et al. 2022) indicate whether each principle was met in this case study and describe the aspects of this project that met each principle (please feel free to refer to Table 1 from the reading for definitions of each principle).

3. Could this template be a useful tool for your team in supporting the development and evaluation of a translational science proposal?

4. What other tools or questions would be useful in evaluation a proposal from a translational science perspective?

01:50
25m

Report-outs and Large Group Discussion

Participants return to main room. Rapporteurs from each group share insights from their group's discussion.

Short facilitated discussion.

ADDITIONAL INFORMATION

Facilitators document common elements of case studies in Jamboard.

02:15
10m

Opportunities and Challenges

Participants share their vision for future shared work in response prompts via Pseudo-Nominal Group Technique.

ADDITIONAL INFORMATION

Prompts:

1. From your vantage point, what is the greatest opportunity this shift in focus to translational science affords us?

2. From your vantage point what is the greatest challenge facing us with the shift in focus to translational science.

Participants submit responses in Jamboard.

02:25
5m

Concluding remarks and next steps

The session closes with a few words from Pillar leadership and an overview by the facilitators and leadership about anticipated next steps.
